# A Randomized Clinical Hypnosis Pilot Study: Improvements in Self-Reported Pain Impact in Adults with Sickle Cell Disease

**DOI:** 10.1155/2021/5539004

**Published:** 2021-08-19

**Authors:** Gwenyth R. Wallen, Kimberly R. Middleton, Narjis B. Kazmi, Li Yang, Alyssa T. Brooks

**Affiliations:** ^1^National Institutes of Health, Clinical Center, Bethesda, MD 20852, USA; ^2^Center for Scientific Review, National Institutes of Health, Division of AIDS, Behavior, and Population Sciences, Bethesda, MD 20817, USA

## Abstract

Sickle cell disease (SCD) is characterized by recurrent painful vasoocclusive crises. Current evidence focuses on the frequency of acute pain crises resulting in emergency department use and nonplanned inpatient hospital admissions; yet few studies focus on pain sequelae outside the healthcare system or how individuals self-manage their chronic SCD-related pain. This study investigated the feasibility of a biobehavioral intervention as an adjunct nonpharmacological therapy to assist in the self-management of chronic pain. A randomized, controlled clinical trial of hypnosis was conducted in outpatients with SCD (*n* = 31). Patient-reported outcomes (PROs) administered at baseline, five, and twelve weeks from both groups included pain frequency, intensity, and quality (Pain Impact Scale (PIQ) and Numerical Rating Scales); anxiety (State-Trait Anxiety Inventory), coping strategies (Coping Strategies Scale), sleep (Pittsburgh Sleep Quality Index (PSQI)), and depression (Beck Depression Inventory (BDI)). The same PROs were collected at weeks seventeen and twenty-four from the control group after the crossover. No significant group by time interaction effects were found in any of the PROs based on the repeated-measures mixed models. The PIQ and PSQI scores decreased over time in both groups. Post hoc pairwise comparisons with the Bonferroni adjustment indicated that the mean PIQ score at baseline decreased significantly by week 12 (*p* = 0.01) in the hypnosis group. There were no significant changes across time before and after the crossover in any of the PROs in the control group. As suggested by these findings, pain impact and sleep in individuals with SCD may be improved through guided mind-body and self-care approaches such as hypnosis.

## 1. Introduction

Sickle cell disease (SCD) is the most common genetic hematologic disease in the United States, characterized by recurrent painful vasoocclusive crises. Sickle cell disease affects approximately 100,000 Americans and with an estimated 1 in 365 African-American newborns each year [1]. The disease is caused by a mutated form of hemoglobin that results in red blood cell (RBC) rigidity, lysis, and clustering. In addition, studies show that the hemoglobin released from intravascular hemolysis scavenges nitric oxide from blood plasma [2], leading to recurrent vasoocclusive crises that are usually accompanied by disabling pain. The severity and frequency of the crises present a significant impact on self-determination, independent living, and overall quality of life [3, 4]. Patients with SCD often report pain, disturbed sleep, reduced daytime functioning, and absence from work or school, all of which can be exacerbated during a vasoocclusive crisis [5, 6]. The standard of care for SCD patients during vasoocclusive crisis is pharmacologic analgesia, typically with opioids. While these pharmacological approaches may be effective for some individuals, many are ineffectively treated due to high dosage requirements, and these approaches do not prevent pain crises from occurring nor ameliorate the consequences of chronic pain in SCD. Evidence often focuses on the frequency of acute pain crises resulting in the need to access emergency departments and/or the number of unplanned inpatient hospitalizations; however, few studies focus on chronic pain manifestations outside the typical healthcare delivery system or how patients self-manage their SCD-related pain. Furthermore, the percentage of patients who are able to self-manage their crises pain and symptoms at home without accessing healthcare professionals is not known [5]. There is growing evidence that the psychosocial and emotional consequences of chronic pain may be modifiable through nonopioid, guided mind-body and self-care approaches such as guided imagery, hypnosis, and yoga; however, it remains unclear whether SCD patients can benefit from these techniques [7–9].

Hypnosis is a cognitive-behavioral intervention that has been shown to have a powerful effect on pain management in a number of acute and chronic settings [10, 11]. Thus, adjunctive treatments using psychosocial methods designed to teach and encourage the use of self-hypnosis may positively impact the pain perception, sleep quality, functional outcomes, quality of life, and satisfaction of individuals with sickle cell, further reducing and/or preventing painful crises and healthcare utilization.

The efficacy of hypnosis has been established in treating numerous conditions including acute pain, chronic pain, burn injury progression, pulmonary illnesses, and hemophilia [12–16]. For those with clinically significant pain episodes, learning a cognitive-behavioral method such as self-hypnosis to manage their pain has proven helpful in reducing pain frequency, improving sleep quality, and decreasing use of opioids [17, 18]. Self-hypnosis training has also been shown to decrease the number of poor sleep nights, mainly by reducing the number of mild-pain nights [18]. Integrating hypnosis and the practice of self-hypnosis into the standard and palliative care of patients with sickle cell disease may also result in better pain management during their crises.

To date, there have been no published randomized, controlled trials evaluating the feasibility/efficacy of hypnosis for pain and symptom control in adults with SCD. This longitudinal clinical trial evaluated the effects of a biobehavioral hypnosis intervention, while assessing the relationships between demographic and psychosocial variables of interest [6]. The primary research objectives of this pilot study were as follows:

  Aim 1: to determine the feasibility of combining heterohypnosis and self-hypnosis as a pain and symptom management strategy in patients with SCD.
  Aim 2: to test whether therapeutic heterohypnosis and self-hypnosis improve disease-related pain, anxiety, coping strategies, sleep, and depression, as compared to an education control intervention in patients with SCD.

## 2. Materials and Methods

### 2.1. Design

**Figure 1:**
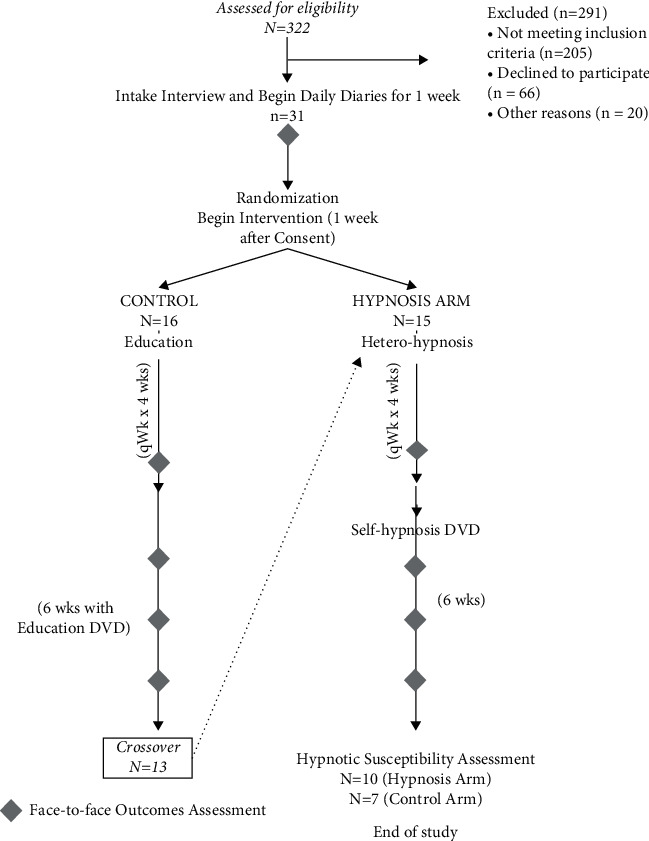
Hypnosis pilot: experimental versus control intervention with SCD patients. Of the 15 participants in Group A (hypnosis arm), 10 participants were considered completers and their data was used in the final analysis. Three participants withdrew (after weeks 0, 1, and 8) and two participants were lost to follow-up (one after week 0 and another after week 5). Of the 16 participants in Group B (control arm), seven completed the study. Six participants withdrew from the study at different time points (weeks 1, 4, 12, 14, and 22), whereas three were lost to follow-up: after week 5, week 14, and week 15.

This was a randomized, controlled, single-crossover, repeated-measures pilot study trial of hypnosis for managing pain in SCD patients (Figure 1). For more details regarding the study design and hypnosis intervention, refer to Wallen et al. (2014) describing the full randomized controlled trial (RCT) protocol [6].

### 2.2. Eligibility

Participants with SCD were recruited into this study by referrals from physicians within one of the Vascular Therapeutic Section, Cardiovascular Branch (CB), National Heart Lung and Blood Institute (NHLBI) at the National Institutes of Health (NIH). Eligibility for this study was limited to hemoglobin SS patients since hemoglobin SC and S-*β*-plus-thalassemia patients typically have less pain. Eligible patients were 18 years of age or older and had a history of pain as a significant problem during a minimum of two days in the month prior to joining the study. Participants provided written informed consent after being provided with the details of the study during an initial face-to-face visit. Participants were excluded from the study if they were unwilling to experience hypnosis or to have heterohypnosis sessions recorded, were nonfluent in written and spoken English, had physical or other disabilities that prevented adequate participation in hypnotic susceptibility testing, did not wish to be video and audiotaped, had psychosis or psychotic depression, and/or had a history of seizures or epilepsy. All participants enrolled in the study received standard-of-care medical therapy while on study irrespective of study group assignment. This standard of care included the full complement of consultations including pain and palliative care services, nutrition services, social workers, spiritual ministry, rehabilitation medicine, and clinical psychiatry.

### 2.3. Instruments

Primary outcome measures included patient assessments of pain frequency, intensity, and quality as measured by the pain numerical rating scale (NRS) on an 11-point scale from 0 to 10, with 0 representing no pain and 10 equaling the worst possible pain [19]. Secondary outcome measures included face-to-face assessments of psychosocial variables including anxiety (Speilberger's State-Trait Anxiety Inventory (STAI)), coping strategies (Coping Strategies Questionnaire (CSQ)), sleep disturbance (Pittsburgh Sleep Quality Index (PSQI)), depression (Beck's Depression Inventory (BDI)), pain impact (The Pain Impact Questionnaire (PIQ-6), and healthcare utilization assessed by unplanned/emergency visits to a hospital, emergency room, or physician's office for crisis pain in the last 24 hours, as reported by patients in their daily diaries. In addition to these patient-reported outcomes (PROs), functional outcomes including ability to work and/or go to school and leave home were also analyzed as part of the daily diaries. Participants were instructed on daily documentation through a Sickle Cell Pain Diary [5, 18, 20] of pain incidence, pain severity, sleep quality, medications taken, and visits to a hospital, emergency room, or physician's office, and absence from school or work. Secondary outcome measures were collected prior to randomization, at the end of the 4-week education or hypnosis interventions, and at two-week intervals until the end of the 6-week self-hypnosis (Group A intervention) or education (Group B control) phases. Detailed description of all study measures is presented in Table 1.

### 2.4. Procedures

Upon consent and enrollment, participants completed a face-to-face intake assessment. After completing the intake questionnaires, each participant was provided with a daily pain diary and instructions on how to complete it for one week. One week following enrollment, participants returned to the outpatient clinic and were randomized to the initial hypnosis intervention group (Group A) or the education control group (Group B) of the study. For more details regarding study enrollment and randomization processes, refer to Wallen et al. describing the full randomized controlled trial (RCT) protocol [6].

Participants in Group A received hypnosis (experimental intervention) during 4 weeks of face-to-face encounters with a physician certified in hypnosis (heterohypnosis). Heterohypnosis sessions consisted of a hypnotic induction followed by individualized suggestions for analgesia, reducing anxiety, improving sleep hygiene, promoting ego-strengthening (self-efficacy), and enhancing health and well-being. Where appropriate, participants also received therapeutic suggestions specific to other symptoms. Sessions lasted about 1-1.5 hours and were typically conducted in a clinic room or other suitable setting. Hypnosis sessions were video and audiotaped for documentation purposes. Following these heterohypnosis sessions in the clinic, participants entered a self-hypnosis phase in which they were trained to perform self-hypnosis. Participants were provided with a DVD for self-hypnosis and a DVD player. For 6 weeks following the instruction period, the participants were instructed to perform self-hypnosis using customizable digital media with a recommended minimum range of three to seven times per week. Participants in the control arm (Group B) of the study received face-to-face education regarding sickle cell disease for the same length and frequency as the treatment group encounters before crossing over to the experimental intervention arm of the study. After completion of the self-hypnosis, an assessment was conducted to measure hypnotic ability, using the *Stanford Hypnotic Susceptibility Clinical Scale for Adults* (SHSS) [33]. This hypnosis susceptibility rating was for documentation purposes and as a potential variable that may be associated with the outcomes of the treatment.

### 2.5. Statistical Analysis

Descriptive statistics (mean and standard deviation for normally distributed continuous data, median for ordinal and nonnormally distributed continuous data, and frequencies and percentages for nominal data) were used to describe the characteristics of the study population and the outcomes (pain, anxiety, coping strategies, sleep, and depression). Correlation matrices and parametric (*t*-test and ANOVA) and nonparametric (Wilcoxon rank-sum and Kruskal–Wallis) tests were used to examine the relationships between the demographic variables and study outcomes at baseline. Overall pain diary measurements, such as percentage of days with SCD pain and other pain, average SCD pain intensity, percentage of days using pain medications during SCD pain days and non-SCD pain days, percentage of bad sleep nights during SCD pain days and non-SCD pain days, and percentage of pain-free days, were computed and compared between two groups using Wilcoxon rank-sum tests and within Group B using Friedman tests. Linear mixed model and Generalized Estimating Equations (GEE) were used to examine the daily SCD pain intensity and bad sleep night's changes over time between two groups.

Linear mixed models for repeated measures were used to examine the changes of the other nondiary outcomes over time before the crossover between the two groups. First-order autoregressive (AR1) and unstructured covariance structures were compared, and model selection was based on Bayesian Information Criterion (BIC). Friedman tests were conducted comparing the differences within Group B across all time points before and after the crossover. Wilcoxon signed-rank tests with a Bonferroni adjustment were conducted to determine pairwise differences. The level of statistical significance was set at 0.05. The data were analyzed using IBM SPSS Statistics and SAS 9.4.

## 3. Results

Out of 117 eligible patients, 31 were enrolled into the study.  Table 2 describes the detailed reasons for exclusion. Of the 31 enrolled, sixteen participants were randomly assigned to the control group and fifteen were assigned to the experimental group. The study participants were 51.6% male with a mean age of 36.2 years; the majority of participants identified themselves as Black/African-American (80.6%) and considered themselves non-Hispanic (93.5%). Majority of the participants (54.8%) completed 75% or more of the pain diaries whereas a little less than half of the participants attended all four heterohypnosis sessions (Table 3). As presented in Tables 4–6 , our data showed no statistically significant differences in selected demographic, baseline clinical characteristics, and clinically relevant lab values and outcome measures between the two groups at baseline.

Using Spearman's rho nonparametric tests for baseline measures, significant correlations were found between NRS and PIQ (rs = 0.412, *p* = 0.021), PIQ and BDI (rs = 0.378, *p* = 0.039), PSQI and BDI (rs = 0.430, *p* = 0.018), BDI and STAI-state (rs = 0.456, *p* = 0.011), STAI-state (rs = 0.456, *p* = 0.011), BDI and STAI-trait (rs = 0.694, *p* < 0.001), STAI-trait and STAI-state (rs = 0.770, *p* < 0.001), STAI-trait and PSQI (rs = 0.361, *p* = 0.046), number of comorbid conditions and number of pain medications (rs = 0.674, *p* < 0.001), and NRS and number of pain medications taken at baseline (rs = 0.382, *p* = 0.041).

At baseline, patients reporting higher levels of pain intensity also reported a higher level of pain severity (rs = 0.412, *p* = 0.021) and greater number of pain medications (rs = 0.382, *p* = 0.041). Similarly, patients reporting higher levels of depression also reported poorer sleep quality (rs = 0.430, *p* = 0.018), higher PIQ (rs = 0.378, *p* = 0.039), higher STAI-trait (rs = 0.694, *p* < 0.001), and higher STAI-state (rs = 0.456, *p* = 0.011). Patients with a higher STAI-trait reported poorer sleep quality (rs = 0.361, *p* = 0.046) and higher STAI-state (rs = 0.770, *p* < 0.001). Patients with a greater number of comorbid conditions reported using a greater number of medications (rs = 0.674, *p* < 0.001).

### 3.1. Primary Outcomes

Linear mixed model for repeated measures for all primary outcomes with AR1 was found to have better fit based on the lower BIC values. No significant group by time interaction effects were found in any of the models (Table 7). Although no group differences were found in any of the primary outcomes, PIQ and PSQI scores decreased significantly over time. Pairwise comparisons (Table 8) using the Bonferroni adjustment indicated that the mean PIQ score at week 12 was significantly lower at week 12 (*p* = 0.003) than at baseline, indicating lower perceived impact of pain following the hypnosis intervention. There was no difference between baseline and week 5 (*p* = 0.11) or between week 5 and week 12 (*p* = 0.20). More specifically, the baseline and week 12 difference was only seen in Group A (*p* = 0.01), the hypnosis intervention group. Group B showed no differences across the three time points. Pairwise marginal means comparison using the Bonferroni adjustment showed that the overall mean PSQI score at week 5 was significantly higher than at week 12 (*p* = 0.04). However, no significant within-group differences were found in the PSQI scores.

Friedman tests examined the outcome measurements in group B over 24 weeks before and after the crossover (Table 9). Only cases that had data for all five time points were used in these analyses. No differences were found for any of these outcomes across time. The authors acknowledge that the lack of effect of the hypnosis intervention in the crossover for the control groups was likely a result of the small sample size.

### 3.2. Diary Outcomes

No significant differences were found in any overall pain diary measurements between two groups in the first ten weeks period and within Group B before and after the crossover (Tables 10 and 11 ).

Using the daily crisis pain intensity as the outcome with random intercept and slope, the linear mixed model showed no significant group, time, and group by time interaction effects.

The Generalized Estimating Equations (GEE) showed no significant group by time interaction and overall group effect in daily bad sleep status. The overall probabilities of bad sleep decreased over time for both groups (*p* = 0.014).

## 4. Discussion

This was the first randomized controlled clinical trial of hypnosis in adults with sickle cell disease that aimed to explore the trajectory of psychosocial variables (depression and anxiety), pain intensity, and pain impact changes over time. Our findings suggest that use of self-hypnosis techniques coupled with heterohypnosis resulted in significant decrease in pain impact and overall improvement in sleep quality over time.

Existing evidence related to the efficacy of hypnosis as a nonpharmacological intervention to address the pain and symptoms often associated with chronic disease management is mixed [34–36]. Heterohypnosis alone or followed by self-hypnosis treatment may benefit some individuals with chronic pain of various etiologies. Previous research has shown that training in different mind-body relaxation techniques, including self-hypnosis, resulted in decrease of emergency room visits and number of hospitalization and inpatient treatment days among patients with a history of painful episodes of sickle cell disease [37]. Dinges et al. reported that self-hypnosis was significantly effective in reducing milder episodes of pain, but not effective in severe sickle cell disease pain episodes while Wolfe and colleagues found that the effects of self-hypnosis on experimental dental pain resulted in increased pain thresholds and lower pain rating on VAS [18, 38].

Elkins et al. highlighted the significant effects of hypnosis on pain reduction in their review of the literature [9, 39–44]. Although our findings did not show a significant reduction in pain as measured by the NRS, our data reflect a significant decrease in pain impact assessed by the Pain Impact Scale (PIQ-6) after a period of 12 weeks. As a validated self-reported measure, the PIQ-6 asks the individual to assess on average how much pain they have had over the past four weeks as well as how this pain has impacted their activities as well as their mood. These findings may be particularly important for SCD patients who not only suffer from episodes of acute pain crisis but also from the physical and emotional sequelae of chronic and often debilitating pain related to their disease. For some patients, reducing their reported current and/or daily perceived pain may not be a realistic goal, whereas building strategies to decrease the negative impact of chronic and crisis pain may be more plausible.

An important aspect of chronic pain is how adversely it affects the individual's overall quality of life. Individuals suffering from SCD crises do require access to medical services for pain control; yet it is important to consider adjunct nonpharmacological strategies for self-care. In the current severe acute respiratory syndrome coronavirus 2 (SARS-CoV-2) pandemic and the opioid crisis eras, self-care modalities that provide individuals with methods to improve sleep and decrease the negative impacts of their chronic pain may be particularly relevant. Self-hypnosis was taught to our intervention group (followed by the education-only group after crossover) in an effort to build a feeling of confidence and self-control over the intensity and frequency of pain. Our results show significant improvement in sleep quality over time, which is consistent with the previous findings of Haanen et al., who also reported improved sleep quality with hypnosis sessions in refractory fibromyalgic pain [9, 40]. Although at baseline, depression and poor sleep quality were significantly correlated in both the intervention and control groups in the current study, and we saw an improvement in sleep quality, our intervention did not result in a significant reduction in depression scores over time. This is in contrast to previous studies that report reductions in fatigue, anxiety, worry, nervousness, and distress with use of hypnotherapy [40, 45, 46]. Future research with a larger sample size is needed to further delineate the effects of hypnotherapy on psychosocial factors in patients with sickle cell disease.

The strength of our study lies in that it addresses the concerns raised by previous hypnosis intervention studies addressing chronic pain and symptom management, namely, the lack of standardization of the hypnotic interventions in clinical trials [9]. Our study is not without limitations in that the results may not be representative of the larger population due to the small sample size and high dropout rates, partially because of the long study duration. Furthermore, future studies may need to consider including symptomatic individuals with hemoglobin SC of Sb + thalassemia in future clinical trials to further evaluate these additional genotypes in adult patients with SCD.

## 5. Conclusions

As suggested by these findings, hypnosis may be a promising tool as an adjunct intervention to reduce pain severity and the impact of pain on an individual's health-related quality of life (HRQOL) as measured by the validated PIQ-6 patient-reported outcome measure which had not been previously used in trials evaluating the use of hypnosis to manage chronic pain in patients with sickle cell disease. Additional randomized trials with larger sample sizes and standardized hypnotic interventions are warranted.

## Figures and Tables

**Table 1:** Description of study measures.

Study measures	Description
Pain Numerical Rating Scale (NRS) [19, 21, 22]	NRS is a numeric version of the visual analog scale in which the participants rate their pain on an 11-point scale. This verbally administered or written scale values range from 0 to 10, with 0 representing no pain and 10 equaling the worst possible pain. The scale has well-established validity.

State-Trait Anxiety Inventory [23–25]	This 40-item inventory assesses two distinct self-report anxiety concepts: state (transitory emotional state) and trait (habitual predisposition to anxiety). Internal consistency coefficients for this scale range from 0.86 to 0.95, whereas reliability and validity have been supported in studies of both patients and normal volunteers.

Coping Strategy Questionnaire (CSQ) [26, 27]	CSQ assesses participant's coping strategies for pain. Individuals rate how often they use each strategy on a 7-point scale for 6 different cognitive/behavioral coping strategies. The scale has acceptable internal reliability

Pittsburgh Sleep Quality Index (PSQI) [28]	PSQI assesses sleep quality and disturbance over a 30-day time interval. 19 individual items generate seven “component” scores and a global score, where a score of 5 or higher indicates poor sleep quality. This scale has been validated in populations with insomnia and other sleep disorders, psychiatric patients, and normal populations. Internal consistency and reliability coefficient range from 0.80 to 0.83 for its seven components.

Beck Depression Inventory (BDI) [29–31]	This 21-item inventory screens for presence and severity of depression in adults. Each item, on a 4-point scale, assesses a particular aspect of depression where higher scores are indicative of more depression. The measure is reliable and valid with adults, including the elderly.

Pain Impact Questionnaire (PIQ) [32]	This 6-item, patient-reported outcome measure assesses pain severity and the impact of pain on an individual's health-related quality of life (HRQOL) over the past four weeks.

Sickle Cell Pain Diary [5]	This pain diary examines painful crises and healthcare utilization events for each participant, noted daily during the study. The sickle cell pain diary included the entire Dinges et al.'s diary with the addition of healthcare utilization items proposed by Smith et al.

**Table 2:** Description of eligible and excluded participants with reasons.

Recruitment status	Total
Eligible	117
Enrolled	31
*Reasons for not enrolling*	
Time constraints	43
Not interested/declined	18
Religion/family obligations	5
Unable to contact	7
Other reasons	13
Not eligible	205
*Reasons for noneligibility*	
Not SS	55
No/limited pain	26
Age, language and comorbidities exclusion	11
Unable to contact	53
Other nonspecific reasons	60

**Table 3:** Participant adherence to study procedures (*n* = 31).

Adherence status	Total *n* (%)	Group A *n* (%)	Group B *n* (%)
*Diary completion*			
25% or less	8 (25.8)	3 (20.0)	5 (31.3)
25%–49%	2 (6.5)	0	2 (12.5)
50%–74%	4 (12.9)	2 (13.3)	2 (12.5)
75% or more	17 (54.8)	10 (66.7	7 (43.8)
*Hypnosis sessions attended*			
None	9 (29.0)	3 (20.0)	6 (37.5)
Two	5 (16.1)	1 (6.7)	4 (25.0)
Three	4 (12.9)	2 (13.3)	2 (12.5)
Four	13 (41.9)	9 (60.0)	4 (25.0)
*Self-hypnosis sessions*			
Minimum and maximum	0–74	7–74	0–56
Median		29	28

**Table 4:** Demographic characteristics of intervention and control group at baseline (*n* = 31).

Characteristics	Total	Group A	Group B
Age			
Mean ± SD	36.2 ± 11.8	37.7 ± 13.6	34.7 ± 10.0
	*n* (%)	*n* (%)	*n* (%)
19–36 years	19 (61.3)	8 (53.3)	11 (68.8)
37–57 years	12 (38.7)	7 (46.7)	5 (31.3)

Gender			
Male	16 (51.6)	6 (40.0)	10 (62.5)
Female	15 (48.4)	9 (60.0)	6 (37.5)

Ethnicity			
Hispanic	2 (6.5)	1 (6.7)	1 (6.3)
Non-Hispanic	29 (93.5)	14 (93.3)	15 (93.8)

Race			
Black/African-American	25 (80.6)	12 (80.0)	13 (81.3)
American Indian/Alaskan Native	1 (3.2)	0 (0)	1 (3.2)
Others	5 (16.1)	3 (20.0)	2 (12.5)

Education			
High school or some college	21 (67.7)	10 (66.7)	11 (68.8)
Graduate or postgraduate	10 (32.3)	5 (33.3)	5 (31.3)

Marital status			
Married	7 (22.6)	3 (20.0)	4 (25.0)
Not married	24 (77.4)	12 (80.0)	12 (75.0)

Comparisons using the independent two-sample *t*-test and Fisher's exact test showed no statistically significant differences for the measures shown.

**Table 5:** Baseline clinical data and clinically relevant lab values (*n* = 31).

	Total	Group A (*n* = 15)	Group B (*n* = 15)
*Clinically relevant lab values* ^*∗*^	Mean (SD) min–max	Mean (SD)	Mean (SD)
Fetal hemoglobin	12.1 (±7.3) 0.0–27.4	14.2 (±8.4)	10.1 (±5.6)
Red blood cell count	2.7 (±0.5) 1.76–3.82	2.7 (±0.5)	2.6 (±0.6)
Hematocrit	26.7 (±3.5) 19.3–34.2	27.2 (±2.4)	26.1 (±4.3)
TR peak velocity (*n* = 27)	2.7 (±0.4) 1.70–3.60	2.7 (±0.5)	2.7 (±0.4)
	***n* (%)**	***n* (%)**	***n* (%)**
*Narcotic analgesics prescribed (baseline)*	26 (89.7%)	11 (78.6%)	15 (100%)
*Episodes of pain in the past 12 months*			
Min and max		0–25	0–20
Median		9.5	4
*Use of hydroxyurea at baseline*	22 (75.9%)	10 (71.4%)	12 (80%)
*Patient reported comorbidities*			
Congestive heart failure	3 (9.7%)	2	1
Chronic lung disease	6 (19.4%)	4	2
Blindness or trouble seeing	4^*∗*^ (12.9%)	4	0
Deafness or difficulty hearing	3 (9.7%)	2	1
Sugar diabetes, mellitus	0	0	0
Asthma	2 (6.5%)	1	1
Ulcer or GI bleeding	1 (3.2%)	0	1
Arthritis or rheumatism	4 (12.9%)	2	2
Sciatic or chronic back pain	6 (19.4%)	3	3
High blood pressure (HTN)	11 (35.5%)	4	7
Angina	2 (6.5%)	2	0
Heart attack or MI	3 (9.7%)	3	0
Stroke	8 (25.8%)	6	2
Kidney disease	3 (9.7%)	3	0
*Number of comorbid conditions*	1.8 (±1.9)	2.4 (±2.2)	1.3 (±1.3)
*Number of medications at baseline*	7.4 (±4.1)	8.4 (±3.9)	6.5 (±4.1)

No statistically significant differences were found between groups based on the independent two-sample *t*-tests. ^*∗*^Normal ranges are fetal haemoglobin = 0.0–2.0; red blood cell count = 3.93–5.22; and haematocrit = 34.1–44.9.

**Table 6:** Primary outcome measures at baseline (*n* = 31).

Outcome measures	Range	Total mean (±SD)	Group A (*n* = 15) Mean (±SD)	Group B (*n* = 16) Mean (±SD)
VAS	0–8	2.7 (±2.3)	3.0 (±2.6)	2.3 (±1.9)
PIQ^*∗*^	48–72	62 (±6.7)	62.8 (±6.5)	61.3 (±7.1)
BDI	0–25	11 (±6.2)	10.7 (±7.0)	11.3 (±5.6)
STAI total	40–117	68.6 (±18.9)	68.2 (±18.8)	69.0 (±19.6)
STAI-state	20–57	32.5 (±9.6)	32.3 (±10.1)	32.8 (±9.4)
STAI-trait	20–60	36.1 (±10.2)	35.9 (±9.5)	36.3 (±11.2)
CSQ	53–209	138.8 (±41.6)	141.1 (±43.7)	136.6 (±40.8)
PSQI	2–16	9.6 (±3.9)	9.2 (±3.6)	10.0 (±4.3)

No statistically significant differences were found between groups based on the independent two-sample *t*-tests. VAS: Visual Analog Scale; PIQ: Pain Impact Questionnaire; BDI: Beck's Depression Inventory; STAI: Speilberger's State-Trait Anxiety Inventory; CSQ: Coping Strategies Questionnaire; PSQI: Pittsburgh Sleep Quality Index. ^*∗*^The US adult general population had an average PIQ score of 50 ± 10; the chronic pain patient sample had a mean score of 64 ± 7 [32].

**Table 7:** Summary of final repeated-measures mixed models of the effect of hypnosis.

Variable	Group		Time		Group^*∗*^time	
	*F* test	*p* value	*F* test	*p* value	*F* test	*p* value
VAS	0.32	0.58	1.83	0.17	0.09	0.92
PIQ	0.17	0.68	6.00	0.005	0.68	0.51
BDI	0.21	0.65	2.02	0.14	0.41	0.67
STAI total	0.80	0.38	0.03	0.98	1.45	0.25
STAI-state	0.57	0.46	0.17	0.84	0.60	0.55
STAI-trait	0.95	0.34	0.93	0.4	2.77	0.07
CSQ	0.84	0.37	0.62	0.54	0.77	0.47
PSQI	0.05	0.82	3.47	0.04	0.56	0.57

Linear mixed models for repeated measures were used to test the hypnosis effect between the two groups over the three time points (baseline, week 5, and week 12). First-order autoregressive covariance structure was used in all models. VAS: Visual Analog Scale; PIQ: Pain Impact Questionnaire; BDI: Beck's Depression Inventory; STAI: Speilberger's State-Trait Anxiety Inventory; CSQ: Coping Strategies Questionnaire; PSQI: Pittsburgh Sleep Quality Index.

**Table 8:** Model estimated means of PIQ and PSQI.

Variable	Group	Baseline	Week 5	Week 12	Overall
PIQ	A	62.8	57.4	53.8	58.0
	B	61.3	59.4	56.2	59.0
	Overall	62.0^*∗*^	58.4	55.0^*∗*^	58.5

PSQI	A	9.2	9.8	7.7	8.9
	B	10.0	9.4	8.1	9.2
	Overall	9.6	9.6^	8.0^	9.0

Model estimated means from the linear mixed models for repeated measures with first-order autoregressive covariance structure. PIQ: Pain Impact Questionnaire; PSQI: Pittsburgh Sleep Quality Index. ^*∗*^Pairwise comparisons using the Bonferroni adjustment indicated mean PIQ score at baseline were significantly higher than at week 12 (*p* = 0.003). ^Pairwise marginal means comparison using the Bonferroni adjustment showed that the overall mean PSQI score at week 5 was significantly higher than at week 12 (*p* = 0.04).

**Table 9:** Group B within group comparisons over time before and after the crossover.

Variable	*N*	*χ*^2^ (d*f* = 4)	*p* value^*∗*^
VAS	7	1.23	0.89
PIQ	7	5.35	0.25
BDI	6	6.63	0.16
STAI total	7	4.65	0.33
STAI-state	7	7.36	0.12
STAI-trait	7	4.98	0.29
CSQ	7	3.25	0.52
PSQI	7	1.63	0.80

VAS: Visual Analog Scale; PIQ: Pain Impact Questionnaire; BDI: Beck's Depression Inventory; STAI: Speilberger's State-Trait Anxiety Inventory; CSQ: Coping Strategies Questionnaire; PSQI: Pittsburgh Sleep Quality Index. ^*∗*^A nonparametric Friedman test was conducted comparing the differences within groups across time.

**Table 10:** Pain diary outcome measurements between two groups.

Variable	Group A median	Group B median	*p* value
Percentage of days with sickle cell disease (SCD) pain	30.95	52.86	0.71
Percentage of days with other pain	56.04	38.96	0.52
Average SCD pain intensity	1.19	1.32	0.85
Percentage of days using pain medications during SCD pain days	99.17	94.12	0.72
Percentage of days using pain medications during non-SCD pain days	29.12	75.00	0.34
Percentage of bad sleep nights (SCD pain days)	24.17	28.42	0.83
Percentage of bad sleep nights (non-SCD pain days)	5.22	15.17	0.39
Percentage of days unplanned ER or doctor visit	3.81	1.45	0.51
Percentage of pain-free days	30.71	16.44	0.60

**Table 11:** Group B within group comparisons for pain diary outcomes over time before and after the crossover.

Variable	Education median	Hypnosis median	*p* value
Percentage of days with sickle cell disease (SCD) pain	52.86	64.29	0.16
Percentage of days with other pain	38.96	39.93	0.71
Average SCD pain intensity	1.32	1.25	>0.99
Percentage of days using pain medications during SCD pain days	94.12	98.11	0.71
Percentage of days using pain medications during non-SCD pain days	75.00	61.34	0.71
Percentage of bad sleep nights (SCD pain days)	28.42	36.31	0.48
Percentage of bad sleep nights (non-SCD pain days)	15.17	14.29	0.10
Percentage of days unplanned ER or doctor visit	1.45	1.92	0.74
Percentage of pain-free days	16.44	11.43	>0.99

## Data Availability

Data are available upon request. Gwenyth Wallen, the Principal Investigator, should be contacted to request the data at gwallen@cc.nih.gov.
